# Non-hematopoietic IL-4Rα expression contributes to fructose-driven obesity and metabolic sequelae

**DOI:** 10.1038/s41366-021-00902-6

**Published:** 2021-07-23

**Authors:** Michelle S. M. A. Damen, Traci E. Stankiewicz, Se-Hyung Park, Robert N. Helsley, Calvin C. Chan, Maria E. Moreno-Fernandez, Jessica R. Doll, Sara Szabo, De’Broski R. Herbert, Samir Softic, Senad Divanovic

**Affiliations:** 1 Department of Pediatrics, University of Cincinnati College of Medicine, Cincinnati, OH USA; 2 Division of Immunobiology, Cincinnati Children’s Hospital Medical Center, Cincinnati, OH USA; 3 Division of Gastroenterology, Hepatology, and Nutrition, Department of Pediatrics, University of Kentucky College of Medicine and Kentucky Children’s Hospital, Lexington, KY USA; 4 Medical Scientist Training Program, University of Cincinnati College of Medicine, Cincinnati, OH USA; 5 Immunology Graduate Program, Cincinnati Children’s Hospital Medical Center and the University of Cincinnati College of Medicine, Cincinnati, OH USA; 6 Division of Pathology, Cincinnati Children’s Hospital Medical Center, Cincinnati, OH USA; 7 Department of Pathobiology, University of Pennsylvania School of Veterinary Medicine, Philadelphia, PA USA; 8 Department of Pharmacology and Nutritional Sciences, University of Kentucky College of Medicine, Lexington, KY USA; 9 Center for Inflammation and Tolerance, Cincinnati Children’s Hospital Medical Center, Cincinnati, OH USA

**Keywords:** Inflammatory diseases, Inflammatory diseases

## Abstract

**Objective:**

The risks of excess sugar intake in addition to high-fat diet consumption on immunopathogenesis of obesity-associated metabolic diseases are poorly defined. Interleukin-4 (IL-4) and IL-13 signaling via IL-4Rα regulates adipose tissue lipolysis, insulin sensitivity, and liver fibrosis in obesity. However, the contribution of IL-4Rα to sugar rich diet-driven obesity and metabolic sequelae remains unknown.

**Methods:**

WT, IL-4Rα-deficient (IL-4Rα^−/−^) and STAT6-deficient mice (STAT6^−/−^) male mice were fed low-fat chow, high fat (HF) or HF plus high carbohydrate (HC/fructose) diet (HF + HC). Analysis included quantification of: (i) body weight, adiposity, energy expenditure, fructose metabolism, fatty acid oxidation/synthesis, glucose dysmetabolism and hepatocellular damage; (ii) the contribution of the hematopoietic or non-hematopoietic IL-4Rα expression; and (iii) the relevance of IL-4Rα downstream canonical STAT6 signaling pathway in this setting.

**Results:**

We show that IL-4Rα regulated HF + HC diet-driven weight gain, whole body adiposity, adipose tissue inflammatory gene expression, energy expenditure, locomotor activity, glucose metabolism, hepatic steatosis, hepatic inflammatory gene expression and hepatocellular damage. These effects were potentially, and in part, dependent on non-hematopoietic IL-4Rα expression but were independent of direct STAT6 activation. Mechanistically, hepatic ketohexokinase-A and C expression was dependent on IL-4Rα, as it was reduced in IL-4Rα-deficient mice. KHK activity was also affected by HF + HC dietary challenge. Further, reduced expression/activity of KHK in IL-4Rα mice had a significant effect on fatty acid oxidation and fatty acid synthesis pathways.

**Conclusion:**

Our findings highlight potential contribution of non-hematopoietic IL-4Rα activation of a non-canonical signaling pathway that regulates the HF + HC diet-driven induction of obesity and severity of obesity-associated sequelae.

## Introduction

Obesity is a major risk factor for development of common, serious medical conditions including low-grade chronic inflammation, insulin resistance, type II diabetes (T2D) and non-alcoholic fatty liver disease (NAFLD) [1]. Over the last few decades, intake of carbohydrates via sugar sweetened food coupled with high-fat (HF) diet consumption has greatly increased. While the general effects of HF diet feeding in experimental models of obesity are well understood, risks of excess sugar (e.g., glucose, sucrose, fructose) intake in addition to HF diet consumption are not well defined. Like HF diet, increased consumption of sugars, specifically high-fructose containing goods, promotes obesity-related sequelae [2]. Both fructose and glucose are 6-carbon sugars, however cellular metabolism of these sugars is divergent. Ubiquitous cellular processing of glucose in the body triggers strong insulin secretion. In contrast, fructose is mainly processed by the liver and only triggers minor insulin secretion. In addition, fructose potentially promotes hepatic triglyceride (TG) accumulation, hepatic de novo lipogenesis (DNL) [3], contributes to (hepatic) insulin resistance [4], and causes mitochondrial dysfunction and inflammation [5, 6].

The immune system is a key pathogenic link between obesity and obesity-associated sequelae [7]. Traditionally, studies have focused on the impact of proinflammatory cytokines (e.g., IL-6, TNF, IL-1, IL-17) while the role of anti-inflammatory cytokines (e.g., IL-4, IL-10, IL-13) in these settings remains insufficiently understood [7, 8]. Further, whether and how fructose impacts anti-inflammatory cytokine production and function in obesity-associated sequelae is not known.

IL-4, a short four α-helix bundle secreted glycoprotein, is a pleiotropic cytokine primarily produced by activated T helper (Th2) cells and eosinophils. IL-4 impacts function of various immune cells including macrophages (e.g., polarization, inflammatory responsiveness), T cells (e.g., expansion, differentiation, cytokine production) and B cells (e.g., isotype class switching from IgM to IgG1 and IgE) [9, 10]. IL-4 signals through its specific type I or type II receptors on the cell surface. The type I receptor consists of 140-kDa binding chain, the α chain (IL-4Rα) and the IL-2 receptor common γ chain (γc) [11]. The type II receptor is established when the IL-4Rα chain forms a complex with the IL-13R chain α1 (IL-13Rα1) [12–14]. The interaction between these two subunits defines IL-4 and IL-13 functional overlap in several biological processes. IL-4 high affinity binding to the IL-4Rα promotes interaction with either the common γ chain or the IL-13R chain α1. As these subunits share equal affinity for the IL-4:IL-4Rα complex the activation of the downstream signaling pathway depends on the specific chain expression on the cell surface [15, 16].

The IL-4RA gene is located on chromosome 16p (16p12.1) in humans and chromosome 7 in mice [17]. IL-4Rα expression is detected in various tissues (e.g., liver, brain, lung, heart, kidney, adipose tissue) and cells (e.g., T cells, B cells, macrophages, epithelial cells, adipocytes). HF diet feeding augmented ex vivo IL-4 secretion by splenocytes [18], white adipose tissue (WAT) inflammation [19], and the severity of obesity-associated sequelae — the latter dependent on IL-4Rα-driven STAT6 activation [20]. IL-4 increases lipolysis by altering hormone sensitive lipase adipogenic/lipolytic activity [21]. Further, it is proposed that IL-4 drives AT browning and energy expenditure (EE) via increased peroxisome proliferator-activated receptor gamma coactivator 1-alpha (PGC-1α) and uncoupling protein 1 (UCP-1) expression in WAT and release of catecholamines by alternatively activated macrophages [22]. However, a subsequent robust mechanistic study demonstrated that alternatively activated macrophages were incapable of synthesizing sufficient amounts of catecholamines to stimulate adipocyte metabolism or thermogenesis and that alterations in the metabolic phenotype of mice could have impacted developmental processes or sympathetic regulation of genes expressed in the nervous system [23].

Given the rapidly increasing prevalence of combined HF and sugar intake and the large diversity in findings on the role of IL-4 in obesity-associated sequelae, we examined the role of IL-4Rα in a combined HF and high carbohydrate/fructose (HC) diet-induced obesity model (HF + HC)– a model that more closely mimics obesity disease. Here we demonstrate that HF + HC diet feeding augmented the occurrence of obesity-associated sequelae via engagement of non-hematopoietic IL-4Rα expression. The effect on weight gain and glucose dysmetabolism in this setting were independent from the canonical IL-4Rα-driven activation of STAT6. IL-4Rα impacted hepatic ketohexokinase-C (KHK-C) and KHK-A expression/activity and correlated with altered hepatic fatty acid oxidation (FAO), fatty acid synthesis (FAS) and hepatic TG accumulation in IL-4Rα-deficient mice fed HF + HC diet. Collectively, our findings demonstrate that the non-hematopoietic IL-4Rα expression is important contributor to combined HF + HC diet-driven obesity.

## Methods

### Mice

All mice used were age-matched males on a C57BL/6 background (Jackson). WT, IL-4Rα deficient (IL-4Rα^−/−^) and STAT6-deficient (STAT6^−/−^) [24] mice were bred at Cincinnati Children’s Hospital Medical Center (CCHMC) in a specific pathogen-free (spf) facility maintained at 22 °C. For the proposed experiments involved, we will use groups sized based on previous studies with HF diet models which suggest that around *n* = 6 mice per group are sufficient to detect statistically significant differences.

### Obesogenic diet model

All diet-induced obesity (DIO) studies were performed as previously described [25–28] with animals being fasted overnight prior to glucose tolerance tests, and terminal harvests. Briefly, 6–8 weeks old mice were started on a diet and were fed either an autoclaved low-fat chow diet food (LAB Diet #5010 (CD); calories provided by carbohydrates (58%), fat (13%) and protein (29%), irradiated high-fat diet (HF; Research Diets #D12492; 20% protein Casein, Lactic, 30 Mesh, Cystine, L; 20% carbohydrate, Lodex 10, Sucrose, Fine Granulated; Fiber; and 60% of calories from fat, Lard, Soybean Oil, USP; Mineral mix S10026B; Vitamin Choline Bitartrate and mix V10001C; energy density 5.21 Kcal/g) or a high-fat high carbohydrate diet (HF Research Diets #D12492i + HC drinking water [HF + HC]). The HC drinking water is a total of 42 g/L of carbohydrates mixed at a ratio of 45% sucrose (Fisher #BP220–1) and 55% fructose (Acros #161350010) by weight, as previously shown [29]. Food and water were replenished weekly. For all studies body weight, food consumption and total caloric intake per mouse were quantified weekly. Total fat mass and lean mass were determined by nuclear magnetic resonance (Whole Body Composition Analyzer; Echo MRI) [30]. In addition, glucose and insulin tolerance tests (ITT) were done as previously described [26]. Energy expenditure (EE) was measured by Phenomaster (TSE systems) as previously described [27]. By having multiple mice in each group data points show biological replicates.

### Quantification of cytokine levels

For quantification of LPS-driven cytokine production in vivo, mice were injected with capture antibodies i.p. 3 h prior to LPS (i.p. 25 μg/mouse) challenge, and serum cytokine levels were determined 4 h later using in vivo cytokine capture assay (IVCCA) as previously described [24, 25, 27, 31].

### mRNA and qPCR analysis

Messenger RNA was extracted as previously described [32]. Quantitative PCR was performed utilizing QuantStudio 7 Real-Time PCR System (Applied Biosystems). Primer sequences used for corresponding genes were (all from 5′ – 3′); *18S* For GTAACCCGTTGAACCCCATT, Rev CCATCCAATCGGTAGTAGCG; *Tbp* For TGACTGCAGCAAATCGCTTGG, Rev ACCCTTCACCAATGACTCCTATG; *Scd-1* For CAGCCGAGCCTTGTAAGTTC, Rev GCTCTACACCTGCCTCTTCG; *Fasn* For GGAGGTGGTGATAGCCGGTAT, Rev TGGGTAATCCATAGAGCCCAG; *Pparα* For GTGGGGAGAGAGGACAGATG, Rev TCCCTGTGAACTGACGTTTG; *Pepck* For TGTCTTCACTGAGGTGCCAG, Rev CTGGATGAAGTTTGATGCCC; *Pgc1α* For AACCACACCCACAGGATCAGA, Rev TCTTCGCTTTATTGCTCCATGA; *TNF* For GATACGTCAAGCCCCTCAAG, Rev CTGCATCAACGTCTTGGAGA; *MCP-1* For TGTCTGGACCCATTCCTTCTTG, Rev AGATGCAGTTAACGCCCCAC; *IL-10* For GAAGCATGGCCCAGAAATCA, Rev TGCTCCACTGCCTTGCTCTT; *F4/80* For CTTTGGCTATGGGCTTCCAGTC, Rev GCAAGGAGGACAGAGTTTATCGTG; *Cpt1a* For AGTGGCCTCACAGACTCCAG, Rev GCCCATGTTGTACAGCTTCC; *Acadvl* For CTGATGAGCTCCCAGGGTAA, Rev TTGGGCCTCTCTAATACCCA; *Acox1* For CCTGATTCAGCAAGGTAGGG, Rev TCGCAGACCCTGAAGAAATC; *Acad9* For CCGACTAGGCCATCTTTTGA, Rev GGAGCTAAAGGGATCTGCAAC; *KHK-C* For AACTCCTGCACTGTCCTTTCCTT, Rev CCACCAGGAAGTCGGCAA; *KHK-A* For TTGCCGATTTTGTCCTGGAT, Rev CCTCGGTCTGAAGGACCACAT; *AldoB* For GCTGGGCAATTTCAGAGAGC, Rev GAGGACTCTTCCCCTTTGCT; *TKFC* For CCTTGCTGGGTTAGTAGCCTC, Rev CTTTCCCGATAAAACCGGCAT.

### Protein extraction and western blot

Liver samples were homogenized in cell lysis buffer containing protease (Bimake, catalog #B14002) and phosphatase inhibitors (Bimake, catalog #15002), as previously described [32]. Proteins were separated using SDS-PAGE and transferred to nitrocellulose membrane (BioRad, catalog #1620112). Immunoblotting was achieved using the following antibodies from Cell Signaling: phospho-Akt (4060), total Akt (4691), phospho-IGF-I/IRβ (3024), total IRβ (3025), phospho-Erk1/2 (4370), and total Erk1/2 (9102). The peroxidase-affinipure goat anti-rabbit (111-035-144) and goat anti-mouse (115-035-003) IgG secondary antibodies were purchased from Jackson ImmunoResearch Laboratories. The vinculin and KHK-C primary antibodies were purchased from Millipore (MAB3574) and Signalway Antibody (21709), respectively. Quantification of immunoblots were performed using ImageJ.

### Hepatic function and phenotyping

Hepatic triglycerides were measured as previously described [5, 33]. For histology, liver tissue was fixed in 10% buffered formalin, stained with H&E. NAFLD activity score (NAS) was determined from H&E staining by a certified pathologist [25–27].

### KHK activity assay

KHK activity was measured using ADP-Glo kinase assay kit (V6930, Promega). Liver tissues were homogenized in 50 mM HEPES, pH 7.4, 150 mM KCl, 4 mM MgCl2, 1 mM Glutathione, 1 mM EDTA, 1 mM DTT, and 0.05% CHAPS using Geno/grinder 2010 (SPEX SamplePrep), and centrifuged for 10 min at 13,000 rpm at 4 °C. The protein supernatant fraction was quantified using the protein BCA assay (Pierce), and KHK activity was measured with 62.5 ng lysate protein after addition of a buffer to 1 mM fructose and 100 µM ATP in 50 mM HEPES, pH 7.4, 150 mM KCl, 4 mM MgCl2, 1 mM Glutathione, and 0.05% CHAPS. After 1-h incubation, ADP-Glo reagent and kinase detection reagent were added with 40 min interval and luminescence was measured using Synergy H1 plate reader (BioTek). KHK activity was normalized using non-protein control group and calculated as fold change with wild type control group.

### BM transfer

BM chimeric mice were generated using 8-week-old WT or IL4Rα-deficient donor and recipient mice and mice were conditioned as previously described [27]. Following successful BM reconstitution, mice were placed on chow, HF + HC diet as described above.

### Mitochondrial isolation and quantification of substrate utilization

Mitochondria were isolated from intrascapular brown adipose tissue (BAT) and processed and analyzed as previously described [27].

### Statistics

For statistical analysis, choice of test was dependent on number of groups and whether normal distribution exists. For all normally distributed data Unpaired two-tailed Student’s *t* test was used to determine differences between groups. Analysis was performed via GraphPad Prism Software’s. Collective indirect calorimetry data was analyzed using analysis of covariance (ANCOVA) with body weight as covariates [34].

### Study approval

All studies were performed in accordance with the procedures outlined in the Guide for the Care and Use of Laboratory Animals and approved by the CCHMC Institutional Animal Care and Use Committee (IACUC).

## Results

### *IL-4Rα* uncouples glucose metabolism from hepatocellular damage

**Fig. 1:**
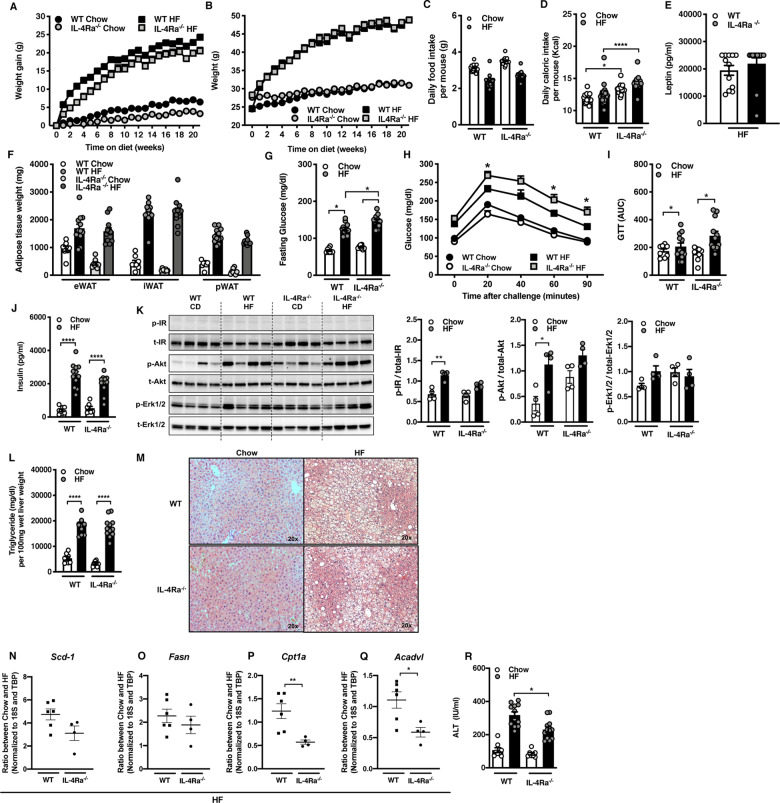
Dichotomous impact of IL-4Rα expression on HF diet-induced metabolic sequelae. WT and IL-4Rα^−/−^ mice were fed a chow or high-fat (HF) diet for 22 weeks. **A** Total body weight gain over time. **B** Absolute body weight over time. **C** Daily food intake per mouse. **D** Daily caloric intake per mouse. **E** Systemic leptin concentrations at time of harvest. **F** White adipose tissue (WAT) weight at time of harvest. eWAT epidydimal WAT, iWAT inguinal WAT, pWAT perirenal WAT. **G** Serum fasting glucose levels at 12 weeks of dietary challenge. **H** Glucose tolerance test at 12 weeks of dietary challenge. **I** Area under the curve (AUC) of glucose tolerance test at 12 weeks of dietary challenge. **J** Fasting serum insulin concentrations at time of harvest. **K** Liver IR, Akt, Erk1/2 levels at time of harvest as determined by western blot. **L** Liver triglycerides at time of harvest. **M** Representative histology images (H&E staining). **N**, **O** Ratios of fatty acid synthesis gene expression in the liver of mice fed HF diet at time of harvest. **P**, **Q** Ratios of Fatty acid oxidation gene expression in the liver of mice fed HF diet at time of harvest. **R** Systemic alanine transaminase (ALT) levels at time of harvest. **A–F** Representative of 2 independent experiments, *n* = 12/condition. **I**, **J** Representative of 2 independent experiments, *n* = 8–12/condition. **K** A single experiment of *n* = 4/condition. **L** Representative of 2 independent experiments, *n* = 8–12/condition. **M** Representative of 2 independent experiments of, *n* = 8–12/condition. **N–Q** A single experiment, *n* = 4/condition. **R** Representative of 2 independent experiments, *n* = 8–12/condition. In bar graphs and line graphs data represents mean ± SEM. **A**, **B**, **N–Q** Unpaired two-tailed Student’s *t* test. *P < 0.05, **P < 0.01, ***P < 0.001, ****P < 0.0001. **H** Area under the curve (AUC). **C–G**, **I–L**, **R** One-way ANOVA followed by multiple comparisons. *P < 0.05, **P < 0.01, ***P < 0.001.

Obesity-associated increase in systemic lipopolysaccharide (LPS) levels contributes to low-grade chronic inflammation and obesity-associated metabolic sequelae [35]. IL-4 via IL-4Rα modulates pro- and anti-inflammatory cytokine production. In vivo LPS challenge of chow diet-fed IL-4Rα-deficient mice, compared to WT controls, drove increased systemic TNF levels and lower IL-10 levels (Supplementary Fig. 1A, B). To correlate the contribution of IL-4Rα altered inflammation to obesity we next studied the role of IL-4Rα using a well-established mouse model of HF diet-induced obesity [36]. In this setting, HF diet-fed IL-4Rα-deficient mice and WT controls exhibited similar body weight (weight gain and absolute), food intake/caloric intake, systemic leptin levels and white adipose tissue (WAT) depot size (Fig. 1A–F).

We next determined the impact of IL-4Rα on severity of obesity-associated metabolic sequelae. HF diet-fed IL-4Rα-deficient mice, compared to WT controls, had increased fasting glucose and impaired glucose tolerance (Fig. 1G–I). This was independent from variable insulin levels and energy expenditure (Fig. 1J, Supplementary Fig. 2A, B). Insulin signaling involves phosphorylation of various protein kinases (e.g., IR, Akt, Erk1/2) to maintain glucose homeostasis [37]. Basal phosphorylation of IR and AKT in liver, which is driven primarily by circulating insulin levels, was upregulated in HF diet-fed WT mice but not IL-4Rα-deficient mice (Fig. 1K), despite increased basal AKT phosphorylation in IL-4Rα-deficient mice fed chow diet. HF diet feeding did not impact Erk1/2 phosphorylation in either WT or IL-4Rα-deficient mice (Fig. 1K).

Hepatic function and triglyceride (TG) accumulation is linked with dysregulated glucose metabolism, increased circulating free fatty acids and increased hepatic fatty acid synthesis (FAS) due to insulin resistance [38]. HF diet-fed IL-4Rα-deficient mice, compared to WT controls, had similar hepatic TG levels (Fig. 1L). HF diet-fed WT mice showed upregulation of essential genes associated with FAS (e.g., *Scd-1*, *Fasn*) compared to low-fat diet-fed counterparts (Supplementary Fig. 3A, B) without altering the expression of FAO genes (e.g., *Cpt1a*, *Acadvl*) (Supplementary Fig. 3C, D). Interestingly, in this setting, IL-4Rα-deficient mice did not increase hepatic expression of FAS-associated genes (e.g., *Scd-1, Fasn*) (Fig. 1N, O). In contrast to HF diet-fed WT mice, IL-4Rα-deficient mice exhibited reduced hepatic expression of FAO-associated genes (e.g., *Cpt1a, Acadvl*) (Fig. 1P, Q). Further, other important genes in FAO (e.g., *Pparα*, *Pgc1α*, *Lcad*, *Acox1)* also tended to be decreased in IL-4Rα-deficient mice (Supplementary Fig. 4A–D). Notably, lack of IL-4Rα expression correlated with mildly reduced hepatocellular damage (Fig. 1M, R). In addition, given the contribution of intestinal inflammation to NAFLD [39], we also examined if the observed effect on obesity-associated sequelae correlated with genotype associated overt intestinal inflammation. Observed effects were independent of genotype associated overt intestinal inflammation (Supplementary Fig. 5). Cumulatively, these findings suggest that IL-4Rα expression uncouples HF diet-associated glucose dysmetabolism from metabolic alterations in the liver.

### *IL-4Rα* regulates carbohydrate diet-driven weight gain and metabolic sequelae

**Fig. 2:**
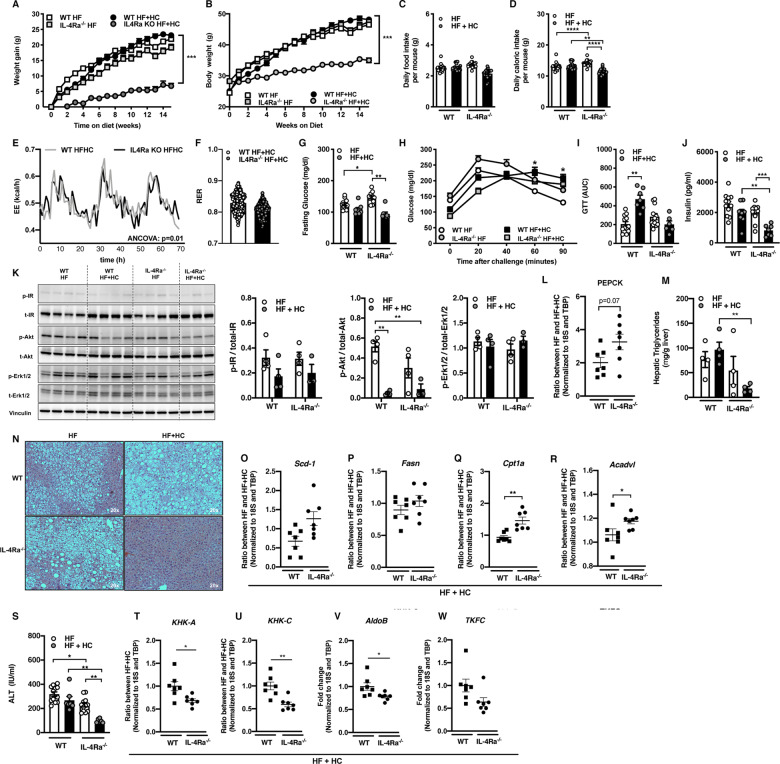
IL-4Rα regulates HF + HC diet-induced weight-gain and associated metabolic sequelae. WT and IL-4Rα^−/−^ mice were fed a HF diet plus supplementation of fructose (HF + HC) for 16 weeks. **A** Total body weight gain over time. **B** Absolute body weight over time. **C** Daily food intake per mouse. **D** Daily caloric intake per mouse. **E** Energy expenditure (EE) in HF + HC at 6 weeks of dietary challenge. **F** Respiratory exchange rate (RER) at 6 weeks of dietary challenge. **G** Serum fasting glucose levels at 12 weeks of dietary challenge. **H** Glucose tolerance test over time at 12 weeks of dietary challenge. **I** AUC of glucose tolerance test at 12 weeks of dietary challenge. **J** Fasting insulin concentrations at time of harvest. **K** Liver IR, Akt, Erk1/2 levels at time of harvest as determined by western blot. **L** PEPCK gene expression in liver at time of harvest. **M**, **N** Liver triglycerides at time of harvest and representative histology images (H&E staining). **O**, **P** Fatty acid synthesis gene expression at time of harvest. **Q**, **R** Fatty acid oxidation gene expression at time of harvest. **S** Systemic alanine transaminase (ALT) levels at time of harvest. **T**, **U** KHK-A and KHK-C expression in liver at time of harvest. **V**, **W** AldoB and TKFC expression in liver at time of harvest. **O–R**, **T–W** Gene expression at time of harvest. **A–D** Representative of 2 independent experiments, *n* = 12/condition. **E**, **F** A single experiment *n* = 4/condition. **G–J** Representative of 2 independent experiments, *n* = 8–12/condition. **K**–**N, O–R** A single experiment *n* = 3–4/condition. **S** Representative of 2 independent experiments *n* = 8–12/condition. **T**–**W** A single experiment *n* = 6/7condition. In bar graphs and line graphs data represents mean ± SEM. **A, B, H** Area under the curve (AUC). **C, D, G, I**–**M, S** One-way ANOVA followed by multiple comparisons. *P < 0.05, **P < 0.01, ***P < 0.001. **D, E** Analysis of covariance (ANCOVA) with body weight as covariate. *p < 0.05, ***p < 0.001. **O–R, T–W** Unpaired two-tailed Student’s *t* test. *P < 0.05, **P < 0.01, ***P < 0.001, ****P < 0.0001.

Consumption of fructose sweetened (high sugar) beverages is considered a key culprit to the obesity pandemic [40]. The role of IL-4Rα in the context of combined high carbohydrate (HC)/fructose beverages and HF diet feeding has not been examined. In WT mice, combined HF + HC feeding compared to HF diet alone yielded similar body weight (weight gain and absolute) (Fig. 2A, B), food/caloric intake (Fig. 2C, D) and systemic leptin levels (Supplementary Fig. 6A). In contrast, weight gain of IL-4Rα-deficient mice was robustly lower under HF + HC conditions (Fig. 2A, B), which was in part independent of food/caloric intake (Fig. 2C, D), water/sugar intake (data not shown [DNS]) or leptin production (Supplementary Fig. 6A). As expected, lower weight gain in IL-4Rα-deficient mice correlated with altered WAT distribution and WAT inflammatory gene expression (Supplementary Fig. 6B, C). IL-4Rα-deficient mice, compared to WT mice fed the HF + HC diet exhibited slight increase in energy expenditure (EE) at hourly rate (Fig. 2E), increased locomotor activity (Supplementary Fig. 6D, E) but similar respiratory exchange rate (RER) (Fig. 2F). Increase in EE was independent of body weight differences (DNS), while alteration to locomotor activity was dependent on body weight (Supplementary Fig. 6E). BAT mitochondrial oxygen consumption rate (OCR) was not altered in WT or IL-4Rα-deficient mice on HF + HC diet (Supplementary Fig. 6F). In addition, the observed effects were independent of genotype associated overt intestinal inflammation (Supplementary Fig. 7) or altered intestinal expression of a glucose transporter GLUT5 (Supplementary Fig. 8).

We next examined the impact of protection from HF + HC diet-induced weight gain on metabolic sequelae. No difference in fasting blood glucose between HF versus HF + HC challenged WT mice was observed. In agreement with lower body weight, IL-4Rα-deficient mice had lower fasting glucose levels on HF + HC diet than on HF diet (Fig. 2G). Further, IL-4Rα-deficient mice, in contrast to WT mice on HF + HC diet, showed a slight reduction in liver specific inflammatory gene expression (Supplementary Fig. 9A) improved glucose and insulin tolerance (Fig. 2H–J and DNS). In addition, HF + HC feeding slightly lowered insulin levels in WT mice however such decrease was significantly more pronounced in IL-4Rα-deficient mice. This was consistent with basal insulin levels (Fig. 2J). Phosphorylation of both the insulin receptor (Fig. 2K) and Akt serine 473 was reduced in context of HF + HC feeding in both genotypes (Fig. 2K). HF + HC diet feeding did not impact Erk1/2 phosphorylation in either WT or IL-4Rα-deficient mice (Fig. 2K). A role of insulin signaling in the liver is to suppress gluconeogenic enzymes via modulation of FoxO1. *PEPCK* gene expression was elevated in HF + HC-fed IL-4Rα-deficient mice (Fig. 2L), consistent with lower basal insulin levels and Akt signaling in these mice.

Protection from weight gain and improved glucose handling in IL-4Rα-deficient mice fed HF + HC diet, compared to WT controls, correlated with decreased hepatic steatosis (Fig. 2M, N). Improved liver TG deposition was independent of altered expression of FAS-associated genes (e.g., *Scd-1* and *Fasn*) (Fig. 2O, P), whereas the expression of FAO genes (e.g., *Cpt1a*, *Acadvl*, *Pgc1α, Acad9*) was increased in IL-4Rα-deficient mice, compared to WT controls (Fig. 2Q, R and Supplementary Fig. 9B, C). In line with reduced hepatic TG levels (Fig. 2M) and increased FAO, IL-4Rα-deficient mice, compared to WT controls, were protected from HF + HC diet-driven hepatocellular damage (Fig. 2S).

Ketohexokinase (KHK) mediates both fructose-induced de novo lipogenesis (DNL) and suppression of FAO [5, 32]. Total KHK is primarily composed of KHK-A (ubiquitously expressed) and KHK-C (primarily expressed in liver, kidney, intestine) subunits. KHK-C exhibits greater affinity for fructose and is expressed ~400x more than KHK-A in the liver (Supplementary Fig. 9D). There was a trend towards reduced expression (Fig. 2T, U) and activity of KHK in IL-4Rα-deficient mice fed HF + HC diet compared to WT controls (Supplementary Fig. 9D–G). Further, other enzymes in fructose metabolism (e.g., AldoB, TFKC) were reduced in IL-4Rα-deficient mice fed HF + HC diet compared to WT controls (Fig. 2V, W). These data suggest an overall reduced ability to metabolize fructose, and may potentially contribute to lower weight gain and improved liver TG handling. Together, our data suggest that lack of IL-4Rα is associated with a trend towards decreased KHK-C expression and increased FAO upon HF + HC diet feeding. Even though HF + HC diet increased overall KHK-activity, reduced KHK-C expression potentially yields lower overall KHK activity in IL-4Rα-deficient mice contributing to metabolic improvements.

### Combination of high carbohydrate with high-fat diet is required for weight gain-associated phenotypes in IL-4Rα-deficient mice

**Fig. 3:**
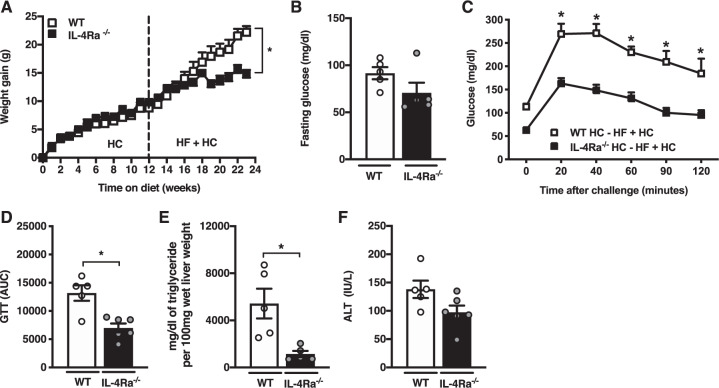
IL-4Rα weight gain-associated sequelae are dependent on the presence of combined high carbohydrate and high-fat diet feeding. WT and IL-4Rα^−/−^ mice were fed a HC diet for 12 weeks and were subsequently switched to a HF or HF + HC diet for an additional of 12 weeks (24 weeks in total). **A** Total body weight gain over time. **B** Serum fasting glucose levels at week 12 of dietary challenge. **C** Glucose tolerance test at 12 weeks of dietary challenge. **D** Area under the curve (AUC) of glucose tolerance test at 12 weeks of dietary challenge. **E** Liver triglycerides at time of harvest. **F** Systemic alanine transaminase (ALT) levels at time of harvest. **A–F** Representative of 2 independent experiments, *n* = 5–6/condition. In bar graphs and line graphs data represents mean ± SEM. **A** Unpaired two-tailed Student’s *t* test. *P < 0.05, **P < 0.01, ***P < 0.001, ****P < 0.0001. **C**, **D** Area under the curve (AUC). **B**, **E**, **F** One-way ANOVA followed by multiple comparisons. *P < 0.05, **P < 0.01, ***P < 0.001.

To determine whether IL-4Rα-associated phenotypic changes were dependent on a HF + HC diet, WT and IL-4Rα-deficient mice were first fed HC diet alone and subsequently switched to a HF + HC diet. Initial exposure to HC did not impact IL-4Rα-dependent modulation of weight gain. Following the diet switch to HF + HC, IL-4Rα-deficient mice were protected from continuous weight gain as compared to WT mice (Fig. 3A). Decreases in weight gain correlated with reduced adiposity, total fat and lean mass (Supplementary Fig. 10A–C) in IL-4Rα-deficient mice fed a HF + HC diet. Fasting blood glucose was slightly decreased in IL4-Rα-deficient mice compared to WT mice, switched to HF + HC diet (Fig. 3B). In agreement with these results, glucose sensitivity was better maintained in IL-4Rα-deficient mice as shown by glucose tolerance testing, compared to WT controls (Fig. 3C, D). The combination of HF + HC feeding also resulted in reduced hepatic TG accumulation and trend towards lower hepatocellular damage in IL-4Rα-deficient mice (Fig. 3E, F). Together, these findings support the importance of IL-4Rα signaling in combined HF + HC diet-induced metabolic sequelae.

### Non-hematopoietic IL-4Rα may contribute obesity and obesity-associated sequelae

**Fig. 4:**
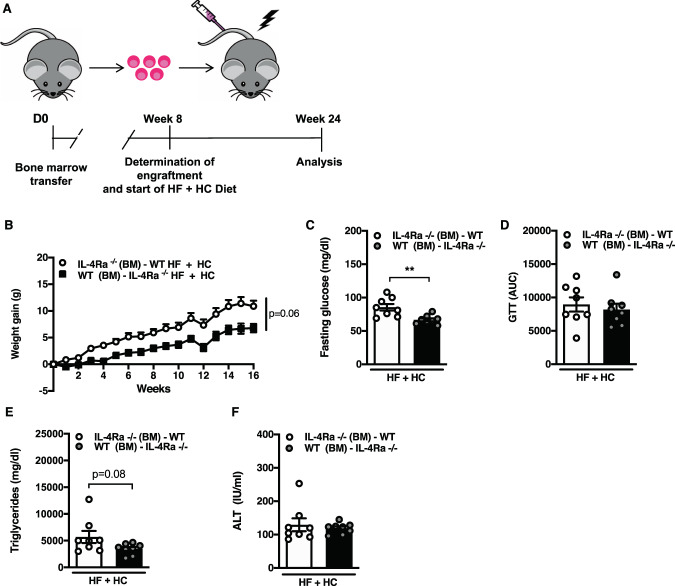
Non-hematopoietic IL-4Rα expression impacts HF + HC induced weight gain and associated sequelae. Reciprocal bone marrow transfers (BMT) between WT and IL-4Rα^−/−^ mice were performed and success of bone marrow reconstitution was confirmed at 8 weeks post-transfer. Successfully transferred mice were subsequently placed on HF + HC diet for 16 weeks. **A** Schematic overview of BMT experimental setup. **B** Total body weight gain over time. **C** Serum fasting glucose levels at 12 weeks of dietary challenge. **D** Area under the curve (AUC) of glucose tolerance test at 12 weeks of dietary challenge. **E** Liver triglycerides at time of harvest. **F** Systemic alanine transaminase (ALT) levels at time of harvest. **B–E** One independent experiment, *n* = 7–8/condition. In bar graphs and line graphs data represents mean ± SEM. **B-F** Unpaired two-tailed Student’s *t* test. *P < 0.05, **P < 0.01, ***P < 0.001, ****P < 0.0001.

IL-4Rα is expressed by various cell types including immune cells, endothelial cells, adipocytes and hepatocytes [41]. As fructose is processed by the liver, we next examined if hematopoietic or non-hematopoietic IL-4Rα was required for modulation of HF + HC diet dependent phenotypes. Reciprocal bone marrow transfers between WT and IL-4Rα-deficient (Fig. 4A) potentially suggested that non-hematopoietic (WT BM to IL-4Rα-deficient mice transfer) locus of IL-4Rα expression was in part responsible for the HF + HC diet-driven weight gain (Fig. 4B) and altered adiposity (Supplementary Fig. 11A–C). The hematopoietic component (BM from WT) which now included IL-4R expression on the hematopoietic cells in IL-4Rα-deficient mice was not sufficient to alter weight gain. In addition, the transfer of IL-4Rα-deficient BM to WT mice did not protect from HF + HC-driven weight gain. Non-hematopoietic IL-4Rα was also involved in regulation of glucose metabolism (Fig. 4C, D). Similarly, hepatic TG accumulation was reduced in mice lacking IL-4Rα expression in non-hematopoietic compartment but no differences were observed in hepatocellular damage (Fig. 4E, F). Together, these findings may invoke the important role of non-hematopoietic locus of IL-4Rα expression as a modulator of HF + HC diet-driven weight gain and obesity-associated sequelae.

### Non canonical IL-4Rα contributes to obesity and metabolic sequelae

**Fig. 5:**
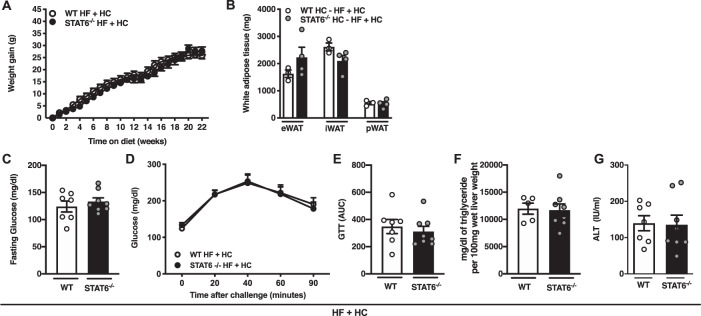
STAT6 does not contribute to combined high-fat and high carbohydrate diet-driven obesity and metabolic sequelae. WT and STAT6^−/−^ mice were fed HF diet plus supplementation of fructose (HF + HC) for 22 weeks. **A** Total body weight gain over time. **B** White adipose tissue (WAT) weight at time of harvest. **C** Serum fasting glucose levels at 12 weeks of dietary challenge. **D** Glucose tolerance test at 12 weeks of dietary challenge. **E** Area under the curve (AUC) of glucose tolerance test at 12 weeks of dietary challenge. **F** Liver triglycerides at time of harvest. **G** Systemic alanine transaminase (ALT) levels at time of harvest. *eWAT* epidydimal WAT, *iWAT* inguinal WAT, *pWAT* perirenal WAT. **A**–**G** One independent experiment, *n* = 7–8/condition. In bar graphs and line graphs data represents mean ± SEM. **A**–**G** Unpaired two-tailed Student’s *t* test. *P < 0.05, **P < 0.01, ***P < 0.001, ****P < 0.0001.

Canonical signaling downstream of IL-4Rα involves STAT6 phosphorylation and induction of various (inflammatory) mediators. IL-4 dependent activation of STAT6 signaling modulates glucose energy metabolism in hematopoietic cells [42]. However, unlike IL-4Rα-deficient mice fed HF + HC diet, STAT6-deficient mice on HF + HC diet were not protected from weight gain (Fig. 5A) and exhibited similar adiposity (Fig. 5B). Further, both WT and STAT6-deficient mice exhibited similar fasting glucose, glucose tolerance (Fig. 5C, D, E), hepatic TG accumulation and hepatocellular damage (Fig. 5F, G). Together, these data suggest that canonical STAT6 signaling is not required to propagate the phenotypes observed in IL-4Rα-deficient mice on HF + HC diet.

## Discussion

The contribution of excessive fructose intake to the growing obesity pandemic and associated pathogenesis of metabolic sequelae remains controversial [43, 44]. Fructose metabolism modulates inflammatory responses and organ and tissue functions [45]. Altered immune responsiveness is a critical causative link between obesity and pathogenesis of obesity-associated diseases. Obesity favors systemic endotoxemia (LPS levels) and systemic inflammation shapes glucose and insulin metabolism [46]. IL-4Rα-deficient mice challenged with LPS exhibited increased proinflammatory cytokine production, invoking the potential role for IL-4Rα-driven regulation of immune responses in glucose metabolism. We show that despite similar degrees of adiposity, IL-4Rα expression uncoupled glucose metabolism from hepatocellular damage in HF diet-fed mice (e.g., reduced hepatocellular damage despite worse glucose dysmetabolism). IL-4 inhibits hepatic expression of PPARα blocking fatty acid beta-oxidation and shifting the metabolic reliance of hepatocytes to glucose oxidation [20]. Downstream catabolic activity of PPARα promotes hepatic and WAT triglyceride oxidation [47]. Given that hepatic PPARα expression was maintained in HF-diet-fed IL-4Rα-deficient mice, these findings could explain the increased hepatic TG accumulation and glucose dysmetabolism but attenuated hepatocellular damage.

In contrast to HF diet feeding, the effect of the IL-4Rα/IL-4/STAT6 axis in combination of HF + HC feeding have not been studied. Combined HF + HC feeding resulted in robust protection from weight gain and weight-associated metabolic derangements in IL-4Rα-dependent manner. Tissue specific inflammatory markers in adipose tissue and liver seemed to be reduced in IL-4Rα-deficient mice compared to WT mice on HF + HC dietary challenge. Dietary challenge-induced systemic and tissue inflammation is associated with increased metabolic sequelae. A reduction in inflammatory markers potentially plays a role in the protection from weight-gain and weight-associated derangements. Published reports suggest that IL-4Rα expression regulates pre-adipocyte differentiation, invoking the possibility of divergent impact of IL-4 on white and brown adipose tissue. AT is a key component to energy expenditure (EE), which in turn regulates body weight. IL-4Rα modified HF + HC-driven EE independent of body weight (Fig. 2E). As the IL-4Rα is a shared receptor for IL-4 and IL-13, altered IL-13 levels in obesity might also play a role. However, HF + HC feeding did not amplify systemic IL-13 levels (data not shown) while IL-4Rα-deficient mice exhibited reduced IL-13 levels (data not shown). Future studies investigating tissue/cell type specific deletion of IL-4Rα in context of HF + HC diet-driven obesity, EE, and metabolic disease are warranted. Analysis of weight matched WT and IL-4Rα groups in context of HF + HC would be central to defining the role of weight gain on the IL-4Rα-dependent metabolic sequelae phenotypes.

Increased hepatic FAO is another mechanism known to regulate weight gain and metabolic derangements [48]. Our data showed upregulated hepatic expression FAO-associated genes in IL-4Rα-deficient mice compared to WT mice fed HF + HC diet. We also showed that HC feeding alone was not sufficient to impact weight gain. A switch to a combined HF + HC diet feeding precluded weight gain in IL-4Rα-deficient mice invoking the possibility that IL-4Rα unlocks a fundamental functional convergence between cellular metabolism and processing of HF + HC diet. Together, these findings amplify the complexity of the IL-4 axis in modulation of obesity and highlight the possibility that observed divergent effects may be dietary substrate driven.

Fructose metabolism primarily takes place in the intestine and liver. Hepatic fructolysis by KHK is associated with high fructose intake-driven weight gain, adiposity, insulin resistance, hyperlipidemia, and DNL [49]. DNL is driven by fructose-mediated insulin signaling independent activation of SREBP-1 and ChREBP-driven lipogenesis [50]. IL-4Rα-deficient mice on HF + HC diet showed improved glucose tolerance and had reduced glucose and insulin levels compared to WT controls. Non-stimulated basal phosphorylation of Akt at serine 473 is driven by circulating insulin levels. Consistent with lower insulin levels, Akt phosphorylation at serine 473 (S473), which impacts protein kinase activity of Akt [51], was lower in IL-4Rα-deficient mice with HF + HC feeding. A role of Akt activation in the liver is to phosphorylate FoxO1, subsequently removing it from the nucleus to lower gluconeognic gene expression. The observed increase in hepatic phosphoenolpyruvate carboxykinase (PEPCK; Fig. 2L) expression in IL-4Rα-deficient mice fed HF and HF + HC diets thus correlates with a decrease in pAkt levels in these mice. Existing literature shows that IL-4Rα recruits the insulin receptor substrate-2 (IRS-2) to associate with PI3K, impacting glucose metabolism that could drive observed differences in insulin signaling in WT versus IL-4Rα-deficient mice [52].

Fructose promotes hepatic steatosis through a variety of mechanisms, including promoting DNL and impairing hepatic FAO [53]. In fact, fructose impairs FAO compared to glucose, whereas KHK knockdown in liver increases FAO gene expression [32]. IL-4Rα-deficient mice fed HF + HC diet exhibited a trend towards decreased protein expression of KHK-C. This suggests that IL-4Rα-deficient mice are not able to sufficiently metabolize fructose in their liver, which could contribute to decreased weight gain, improved steatosis and better glucose tolerance. In addition, a decrease in liver KHK-C expression correlated with a concomitant increase in the expression of FAO-associated genes compared to WT mice in line with our previous result that knockdown of KHK increased hepatic FAO [32]. Thus, potential IL-4Rα-driven regulation of KHK-C and hepatic FAO could in part explain the reduced hepatic TG levels and hepatocellular damage.

Aside from immune cells, various non-hematopoietic cells and tissues express IL-4Rα. We show that non-hematopoietic expression of IL-4Rα potentially contributed to the protective phenotype observed upon HF + HC diet feeding. Transfers of WT bone marrow into IL-4Rα-deficient recipient mice yielded reduced weight gain, adiposity, fasting glucose levels, hepatic TG levels, and hepatocellular damage. Expression of IL-4Rα in non-hematopoietic cells contributed to other diseases including asthma, cancer, and hepatitis [54, 55]. IL-4Rα drives hepatic apoptosis, myofibroblast differentiation, and pre-adipocyte differentiation. Thus, alterations in non-hematopoietic IL-4Rα expression may impact hepatocellular damage, hepatic fibrosis, and adipocyte differentiation and function. Future studies fully warrant the investigation of tissue specific differences in non-hematopoietic IL-4Rα expression and their contribution to HF- and HF + HC diet-induced obesity.

Activation of STAT6 impacts cell polarization and differentiation and regulates immune responses [56]. IL-4 and IL-13 are two main drivers of STAT6 activation. Where the contribution of STAT6 in HF diet feeding has been demonstrated [20] the role of STAT6 in HF + HC diet feeding remained undefined. Unlike the observed protection from weight gain in IL-4Rα-deficient mice on HF + HC diet, STAT6 KO mice on HF + HC diet were not protected from weight gain, adiposity, glucose dysmetabolism, or hepatocellular damage. Importantly, tissue specific differences in STAT6 expression between the liver, skeletal muscle, and AT could contribute to observed differences in metabolic derangements [57]. As a number of non-canonical pathways can drive IL-4 mediated effects [58] our data also suggest that activation of a non-canonical pathway (e.g., Notch, mTOR) [59, 60] downstream of IL-4Rα may be responsible for observed phenotypes. The contribution of these pathways to HF and HF + HC induced obesity is warranted.

**Fig. 6:**
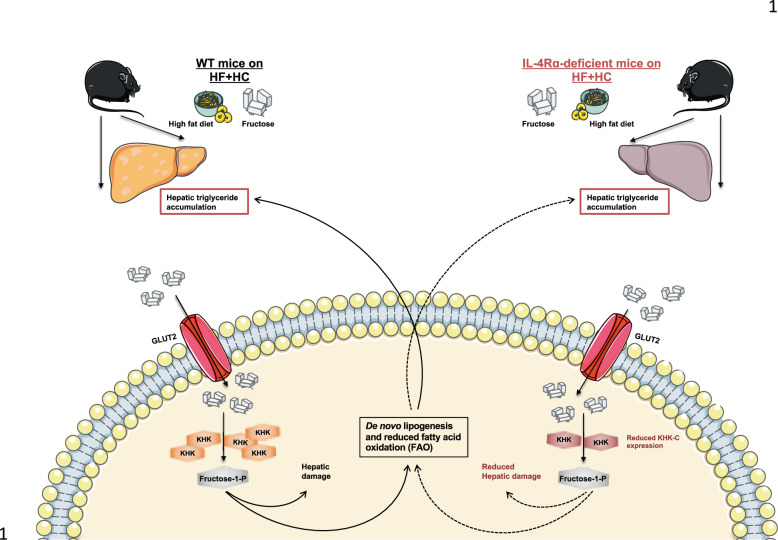
Schematic overview of potential IL-4Rα contribution to HF + HC-driven obesity and obesity-associated metabolic derangements. Schematic overview of the potentially impact of IL-4Rα-deficiency on hepatic fructose metabolism in mice fed high-fat + high carbohydrate diet. IL-4Rα-deficient mice on HF + HC diet show protection from weight gain, adiposity, insulin dysmetabolism and hepatocellular damage. Fructose is metabolized in the intestine and the liver. KHK-C is the first/key enzyme in fructolysis. KHK-C impacts de novo lipogenesis (DNL) and suppress fatty acid oxidation (FAO). Our data shows that IL-4Rα-deficient mice potentially have lower KHK-C expression which can explain their increased expression of genes related to FAO and could represent an underlying mechanism explaining their protection from weight gain and associated metabolic derangements.

In sum, our report highlights an undervalued role for IL-4Rα signaling in the context of high fructose-driven obesity suggesting that greater examination of this signaling pathway could lead to novel insights into disease pathogenesis (Fig. 6). Thus, it is plausible that novel pharmacological intervention in the IL-4/IL4Rα axis function, in both hematopoietic as well as non-hematopoietic cells, would provide novel approaches to dampen high dietary fructose-driven metabolic harm associated with obesity.

### Study limitations

Our new insights demonstrating the impact of IL-4Rα expression to high fructose-driven obesity development provide the opportunity for greater examination of IL-4 axis and the downstream linked non-canonical pathways to high-fat + high carbohydrate diets-associated metabolic disease pathogenesis. Our study however still leaves several questions unanswered. Specifically, future mechanistic interrogations of the pathways/signaling cascades underlying IL-4Rα expression importance in this setting are needed. Our studies also have not examined whether the observed effects are gender specific given previous reports invoking the importance of thermoneutral housing and hormonal imbalance on HF diet-driven metabolic sequelae in female mice. Additionally, future studies should focus on analysis of weight matched WT and IL-4Ra^−/−^ mice- findings central for uncovering the impact of weight difference on metabolic disease severity in this system. We also did not examine the impact of tissue specific deletion of IL-4Rα or STAT6 expression and whether specific tissues/cell types could dichotomously impact inflammatory/anti-inflammatory responses in this setting and thus shape metabolic sequelae. Lastly, our study did not exploit potential differences in systemic inflammatory phenotypes and immune cell development and function between the HF and HF + HC-fed groups. Recent studies have shown that proinflammatory mediators may regulate metabolic sequelae [61]. Further, specific hepatic inflammatory Th17 cells specifically contribute to obesity induced liver damage [62]. Combined future investigations in these areas will be critical to pinpoint the interplay of immune cells and tissue non-hematopoietic cells utilizing IL-4Rα-dependent signaling in the setting of HF versus HF + HC diet exposure and the relevance of such biological processes to obesity-driven metabolic disease progression.

## Supplementary information

Supplementary Figure legends

Supplementary Figure 1

Supplementary Figure 2

Supplementary Figure 3

Supplementary Figure 4

Supplementary Figure 5

Supplementary Figure 6

Supplementary Figure 7

Supplementary Figure 8

Supplementary Figure 9

Supplementary Figure 10

Supplementary Figure 11
