# Supplementary material for: Non-hematopoietic IL-4Rα expression contributes to fructose-driven obesity and metabolic sequelae
Source: Int J Obes (Lond). 2021 Jul 23;45(11):2377–87. doi: 10.1038/s41366-021-00902-6 (PMC8528699; doi:10.1038/s41366-021-00902-6)
Supplement: Supplementary file 1 — Supplementary Figure legends [file 41366_2021_902_MOESM1_ESM.docx]

**Supplementary Figure 1.** **IL-4Rα** **impacts baseline systemic inflammation.** WT and IL-4Rα^-/-^ mice fed chow diet for 22 weeks where challenged with LPS in the presence of IVCCA antibodies and cytokine production was analyzed by IVCCA-ELISA 4 hours after challenge. (**A**) Systemic TNF levels. (**B**) Systemic IL-10 levels. (**A-B**) One independent experiment, n = 6-8/condition. In bar graphs and line graphs data represents mean +/- SEM. (**A-B**) Unpaired two-tailed student’s t-test. *P < 0.05, **P < 0.01, ***P < 0.001, ****P < 0.0001.

**Supplementary Figure 2. IL-4Rα expression does not contribute to changes in energy expenditure in chow or HF diet fed mice.** Extended data from Figure 1. WT and IL-4Ra^-/-^ mice were fed chow or high fat (HF) diet for 16 weeks. (**A**) Energy expenditure (EE) in chow fed mice at week 6 of dietary challenge. (**B**) Energy expenditure (EE) in HF diet fed mice at week 6 of dietary challenge. (**A-B**) A single experiment of n = 3-4/condition. Analysis of covariance (ANCOVA) with body weight as covariate.

**Supplementary Figure 3. HF diet feeding impacts fatty acid synthesis in WT mice.** Extended data from Figure 1. WT mice were fed chow or HF diet for 22 weeks. (**A-B**) Ratios of FAS-associated gene expression in liver at time of harvest. (**A**) *Scd-1*. (**B**) *Fasn..* (**C-D**) Ratios of FAO-associated gene expression in liver at time of harvest. (**C**) *Cpt1a*. (**D**) *Acadvl*. (**A-D**) A single experiment of, n = 4/condition. In graphs data represents mean +/- SEM. Unpaired two-tailed student’s t-test. *P < 0.05, **P < 0.01, ***P < 0.001, ****P < 0.0001.

**Supplementary Figure 4. HF diet feeding may alter expression of fatty acid oxidation genes in IL-4Rα-deficient mice.** Extended data from Figure 1. WT and IL-4Rα^-/-^ mice were fed HF diet for 22 weeks. (**A-D**) Ratios of FAO-associated gene expression in liver at time of harvest. (**A**) *Pparα*. (**B**) *Pgc1α*. (**C**) *Lcad*. (**D**) *Acox1*. (**A-D**) A single experiment of, n = 4/condition. In graphs data represents mean +/- SEM. Unpaired two-tailed student’s t-test. *P < 0.05.

**Supplementary Figure 5. Intestinal histology of WT and IL-4Rα-deficient mice on chow or HF diet.** Extended data from Figure 1. WT and IL-4Rα^-/-^ mice were fed HF diet for 22 weeks. H&E staining of intestines from WT and IL-4Rα^-/-^ at time of harvest (magnification 20x).

**Supplementary Figure 6. IL-4Rα expression contributes to HF+HC diet weight-gain and obesity-associated metabolic sequelae.** Extended data from Figure 2. WT and IL-4Ra^-/-^ mice were fed HF diet plus supplementation of fructose (HF+HC) diet for 16 weeks. (**A**) Systemic leptin levels at time of harvest. (**B**) White adipose tissue (WAT) weight at time of harvest. eWAT = epidydimal WAT; iWAT = inguinal WAT; pWAT = perirenal WAT. (**C**) eWAT inflammatory gene expression between HF and HF+HC based on fold change over WT HF expression. (**D-E**) Locomotor activity at week 6 of dietary challenge. (**F**) Oxygen consumption rate (OCR) measured in BAT at time of harvest. (**A-B, F**) Representative of 2 independent experiments, n = 12/condition. (**C-E**) A single experiment, n = 3-4/condition. In bar graphs and line graphs data represents mean +/- SEM. (**A-C, E-F**) Unpaired two-tailed student’s t-test. *P < 0.05, **P < 0.01, ***P < 0.001, ****P < 0.0001. (**D-E**) Analysis of covariance (ANCOVA) with body weight as covariate. *p < 0.05, ***p < 0.001.

**Supplementary Figure 7. Intestinal histology of WT and IL-4Rα-deficient mice on HF or HF+HC diet.** Extended data from Figure 2. WT and IL-4Ra^-/-^ mice were fed HF diet plus supplementation of fructose (HF+HC) diet for 16 weeks. H&E staining of intestines from WT and IL-4Rα^-/-^ at time of harvest (magnification 20x).

**Supplementary Figure 8. Intestinal expression of GLUT5 in WT and IL-4Rα-deficient mice on HF or HF+HC diet.** Extended data from Figure 2. WT and IL-4Ra^-/-^ mice were fed HF diet plus supplementation of fructose (HF+HC) diet for 16 weeks. Glut5 expression and quantification in intestine at time of harvest.

**Supplementary Figure 9. IL-4Rα signaling potentially contributes to reduced inflammatory gene expression in liver**. Extended data from figure 2. WT and IL-4Ra-/- mice were fed HF diet or HF plus supplementation of fructose (HF+HC) diet for 16 weeks. (**A**) Liver tissue specific inflammatory gene expression between HF and HF+HC based on fold change over WT HF expression. (**B-C**) Fatty acid oxidation gene expression in liver at time of harvest. (**C-F**) KHK-C protein expression and quantification in liver at time of harvest. (**G**) KHK activity in liver at time of harvest. (**A**) A single experiment, n = 3-4/condition. (**B-C**) Representative of 2 independent experiments, n = 12/condition. (**D-G**) A single experiment, n = 3-4/condition. In bar graphs and line graphs data represents mean +/- SEM. (**B-G**) Unpaired two-tailed student’s t-test. *P < 0.05, **P < 0.01, ***P < 0.001, ****P < 0.0001.

**Supplementary Figure 10. IL-4Rα effects on weight gain are dependent on HF+HC dietary challenge**. Extended data from Figure 3. WT and IL-4Rα^-/-^ mice were fed an HC diet for 12 weeks and were subsequently switched to an HF or HF+HC diet for an additional of 12 weeks (24 weeks in total). (**A**) White adipose tissue (WAT) weight at time of harvest. eWAT = epidydimal WAT; iWAT = inguinal WAT; pWAT = perirenal WAT. (**B**) Percentage of fat mass per group of mice at 23 weeks of dietary challenge. (**C**) Percentage of lean mass per group of mice at 23 weeks of dietary challenge. (**A-C**) Representative of 2 independent experiments, n = 5-6/condition. In bar graphs and line graphs data represents mean +/- SEM. (**A-C**) Unpaired two-tailed student’s t-test. *P < 0.05, **P < 0.01, ***P < 0.001, ****P < 0.0001.

**Supplementary Figure 11. Non-hematopoietic IL-4Rα expression partly alters HF+HC diet driven WAT distribution.** Extended data from Figure 4. Reciprocal bone marrow transfers (BMT) between WT and IL-4Rα^-/-^ mice were performed and success of bone marrow reconstitution was confirmed at 8 weeks post-transfer. Successfully transferred mice were subsequently placed on HF+HC diet for 16 weeks. (**A**) eWAT = epidydimal weights at time of harvest. (**B**) iWAT = inguinal WAT weights at time of harvest. (**C**) pWAT = perirenal WAT weights at time of harvest. (**A-C**) One independent experiment, n = 7-8/condition. In bar graphs and line graphs data represents mean +/- SEM. (**A-C**) Unpaired two-tailed student’s t-test. *P < 0.05, **P < 0.01, ***P < 0.001, ****P < 0.0001.
