# Supplementary material for: Non-hematopoietic IL-4Rα expression contributes to fructose-driven obesity and metabolic sequelae
Source: Int J Obes (Lond). 2021 Jul 23;45(11):2377–87. doi: 10.1038/s41366-021-00902-6 (PMC8528699; doi:10.1038/s41366-021-00902-6)
Supplement: Supplementary file 2 — Supplementary Figure 1 [file 41366_2021_902_MOESM2_ESM.pdf]

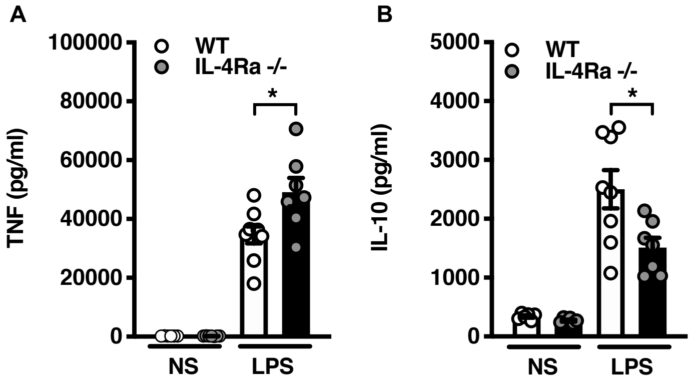

Supplementary Figure 1. IL-4Rα impacts baseline systemic inflammation. WT and IL-4Rα$^{-/-}$ mice fed chow diet for 22 weeks where challenged with LPS in the presence of IVCCA antibodies and cytokine production was analyzed by IVCCA-ELISA 4 hours after challenge. (**A**) Systemic TNF levels. (**B**) Systemic IL-10 levels. (**A-B**) One independent experiment, n = 6-8/condition. In bar graphs and line graphs data represents mean +/- SEM. (**A-B**) Unpaired two-tailed student's t-test. *P < 0.05, **P < 0.01, ***P < 0.001, ****P < 0.0001.
