# Supplementary figures and images for: Non-hematopoietic IL-4Rα expression contributes to fructose-driven obesity and metabolic sequelae
Source: Int J Obes (Lond). 2021 Jul 23;45(11):2377–87. doi: 10.1038/s41366-021-00902-6 (PMC8528699; doi:10.1038/s41366-021-00902-6)

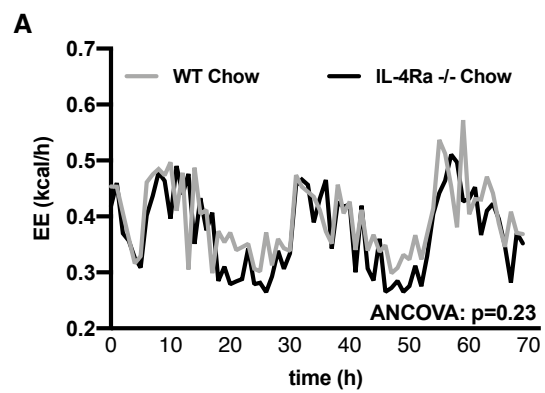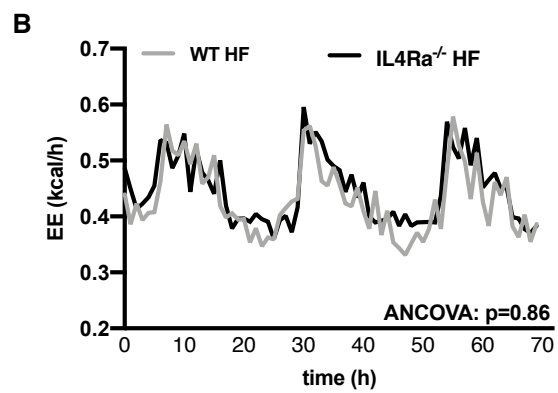

Supplement: Supplementary file 3 — Supplementary Figure 2 [file 41366_2021_902_MOESM3_ESM.pdf]

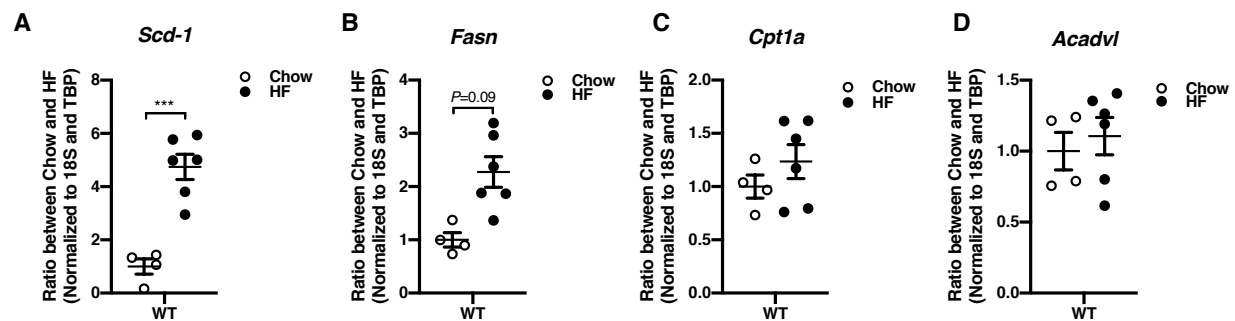

Supplement: Supplementary file 4 — Supplementary Figure 3 [file 41366_2021_902_MOESM4_ESM.pdf]

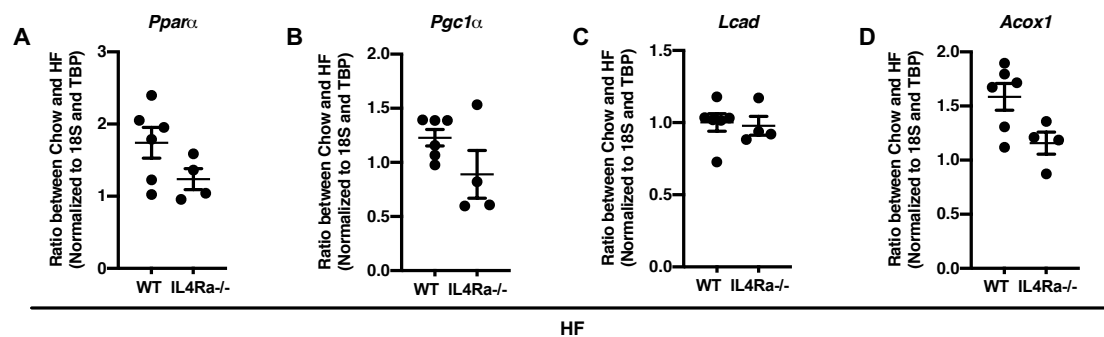

Supplement: Supplementary file 5 — Supplementary Figure 4 [file 41366_2021_902_MOESM5_ESM.pdf]

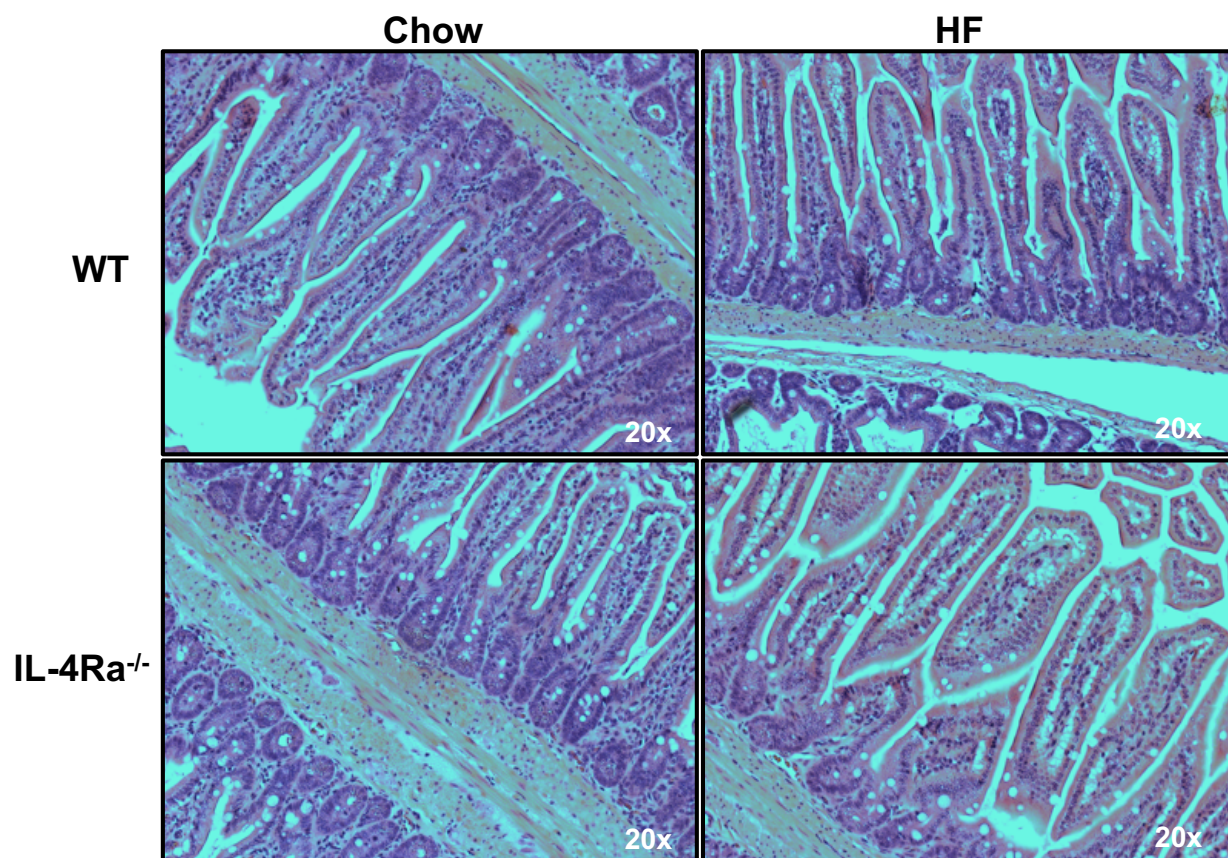

Supplement: Supplementary file 6 — Supplementary Figure 5 [file 41366_2021_902_MOESM6_ESM.pdf]

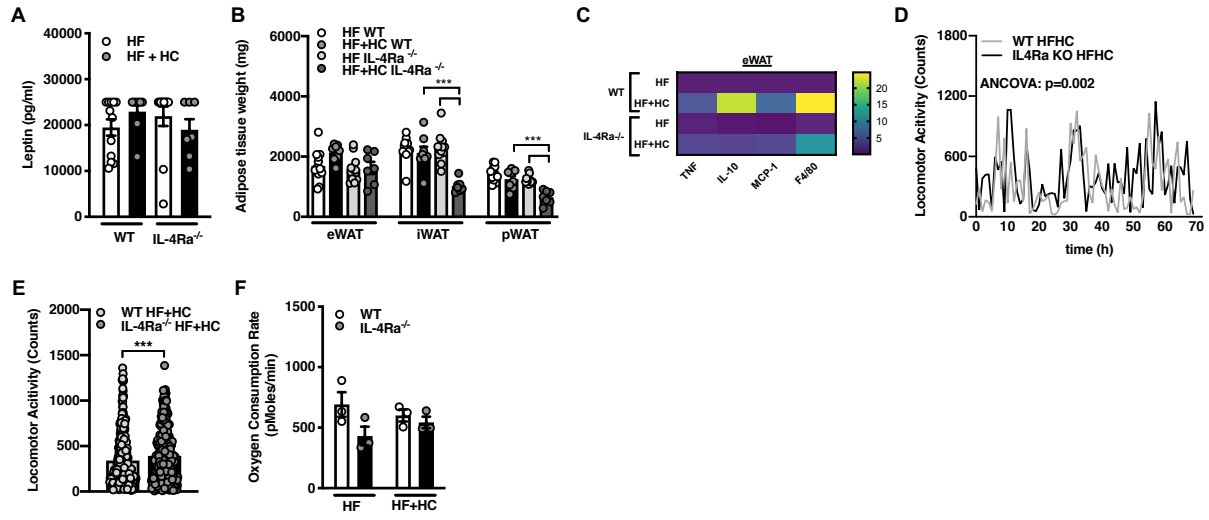

Supplement: Supplementary file 7 — Supplementary Figure 6 [file 41366_2021_902_MOESM7_ESM.pdf]

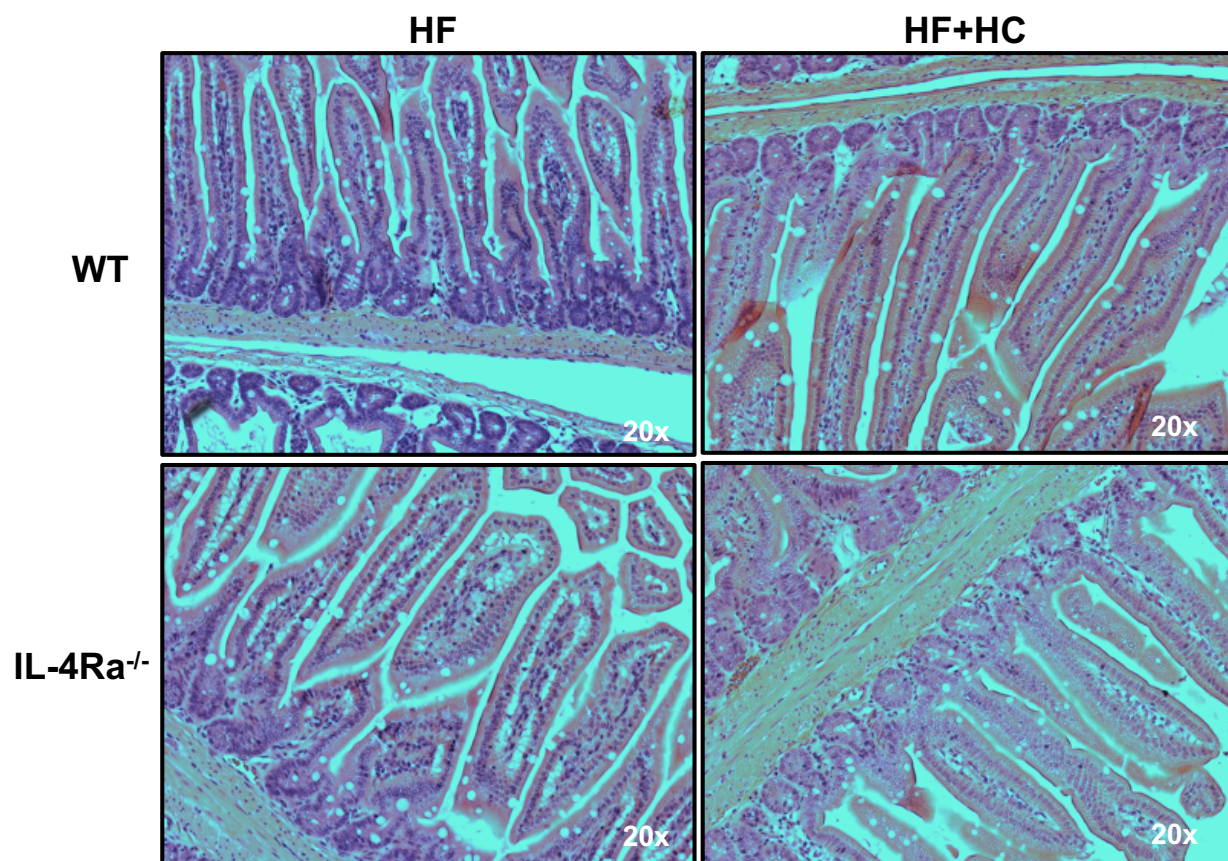

Supplement: Supplementary file 8 — Supplementary Figure 7 [file 41366_2021_902_MOESM8_ESM.pdf]

**GLUT5**

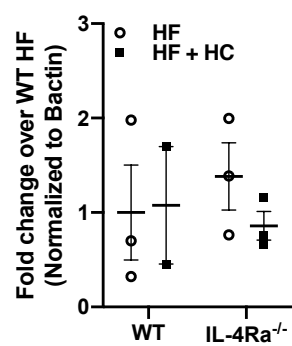

Supplement: Supplementary file 9 — Supplementary Figure 8 [file 41366_2021_902_MOESM9_ESM.pdf]

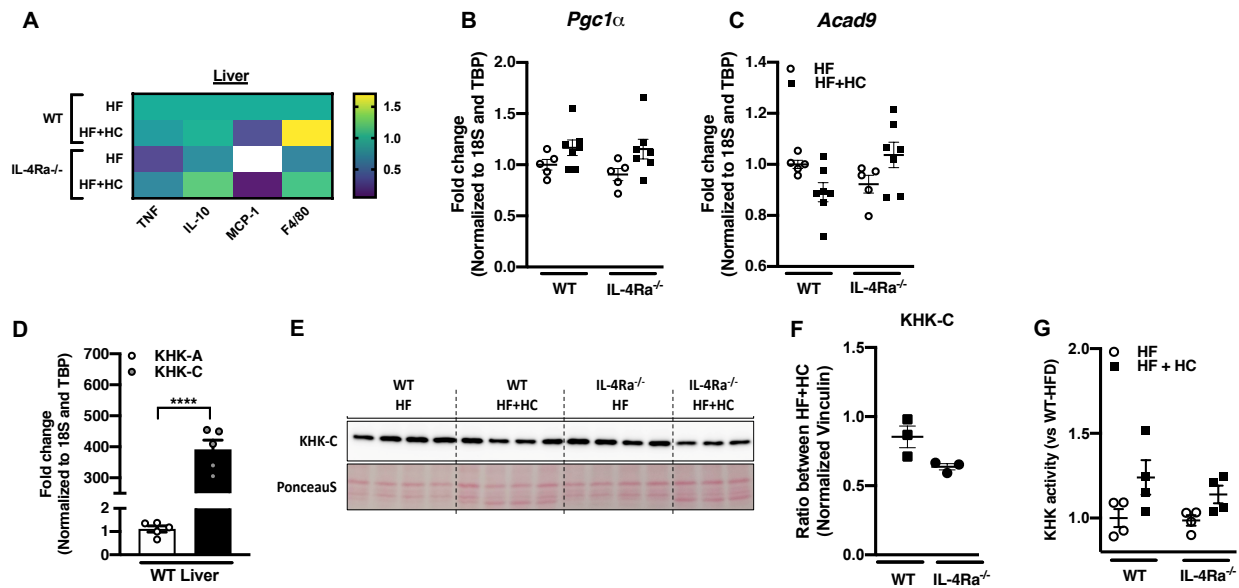

Supplement: Supplementary file 10 — Supplementary Figure 9 [file 41366_2021_902_MOESM10_ESM.pdf]

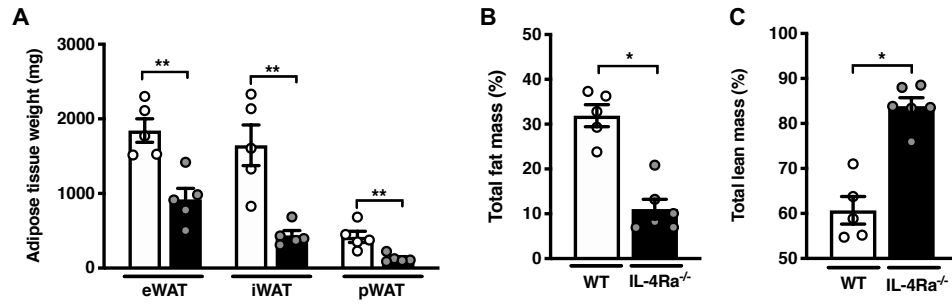

Supplement: Supplementary file 11 — Supplementary Figure 10 [file 41366_2021_902_MOESM11_ESM.pdf]

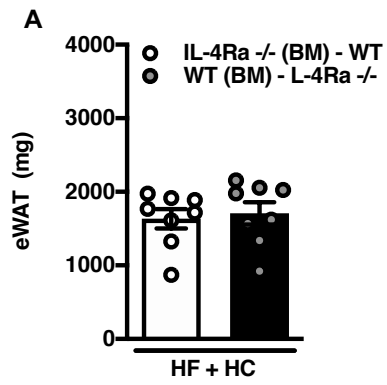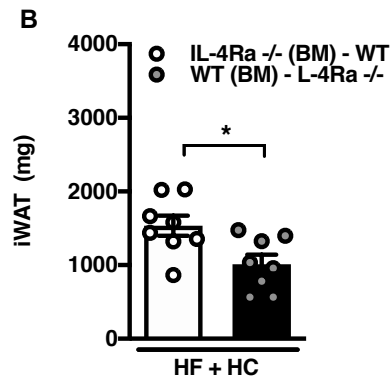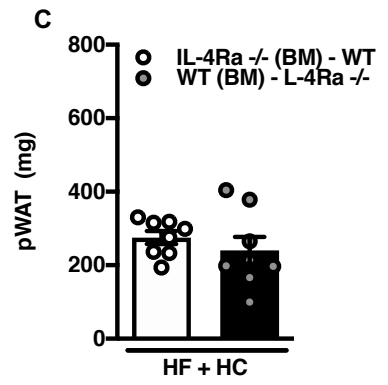

Supplement: Supplementary file 12 — Supplementary Figure 11 [file 41366_2021_902_MOESM12_ESM.pdf]
